# Hypereosinophilia and eosinophilic arterial aneurysm: not only eosinophilic granulomatosis with polyangiitis

**DOI:** 10.1093/rap/rkae138

**Published:** 2024-11-08

**Authors:** Elena Vanni, Chiara Marvisi, Carlo Salvarani

**Affiliations:** Rheumatology Unit, Azienda USL-IRCCS of Reggio Emilia and University of Modena and Reggio Emilia, Reggio Emilia, Italy; Rheumatology Unit, Azienda USL-IRCCS of Reggio Emilia and University of Modena and Reggio Emilia, Reggio Emilia, Italy; Rheumatology Unit, Azienda USL-IRCCS of Reggio Emilia and University of Modena and Reggio Emilia, Reggio Emilia, Italy

A 61-year-old Caucasian female was diagnosed with Eosinophilic Granulomatosis with Polyangiitis (EGPA) in 2015 for allergic asthma, nasal polyposis, mild eosinophilia and sporadic paresthesias. She also presented a right ulnar artery aneurysm (Fig. 1A–C), which was excised and confirmed histologically compatible with EGPA. Following a short course of glucocorticoids, she began low-dose methotrexate.

**Figure 1.**
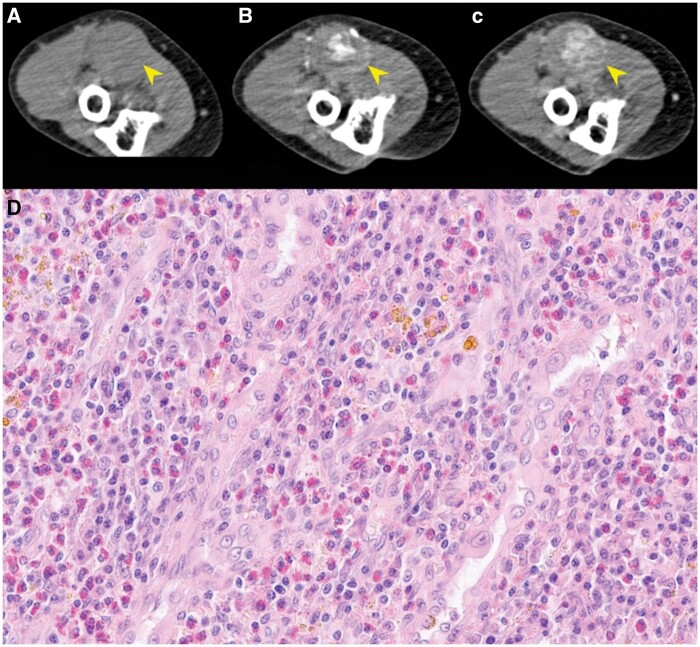
CT appearance of the ulnar artery aneurysm and histopathological findings compatible with AHLE. (A–C): CT scan showing the right ulnar artery aneurysm (arrows) in the precontrast phase (A), arterial phase (B) and portal venous phase (C). (D) Histologic finding at biopsy, showing small vessel lined by epithelioid endothelium and a dense perivascular eosinophilic infiltrate. Hemosiderin pigment is present in the infiltrate. Haematoxylin–Eosin 200×.

She was then referred to our centre for a second opinion. Upon admission, a comprehensive diagnostic workup, including electromyography, brain MRI, pulmonary and maxillofacial CT scans and urine sediment analysis, revealed no active disease. ANCAs were negative and asthma was well-controlled. BVAS was 0.

Histological specimens were re-evaluated, revealing small vessels lined by plump, epithelioid endothelial cells and a dense perivascular eosinophilic infiltrate (Fig. 1D). No fibrinoid necrosis or eosinophilic granulomas were present.

Given the benign clinical course and histological findings, a diagnosis of angiolymphoid hyperplasia with eosinophilia (ALHE) was established and methotrexate was stopped without subsequent relapses.

ALHE is a rare angioproliferative disorder typically affecting middle-aged women, presenting mainly as dermal papules or nodules, with generally absent systemic involvement. Histologically, it features proliferative capillary vessels with epithelioid-like cells and a variable lymphocytic and eosinophilic infiltrate [1]. Unlike EGPA, ALHE has a benign prognosis, often with spontaneous remission and infrequent postsurgical recurrences [2]. Correct diagnosis is crucial to avoid unnecessary immunosuppressive treatments.

## Data Availability

The data underlying this article will be shared on reasonable request to the corresponding author.
